# Supplementary material for: Leveraging computerized adaptive testing for cost-effective evaluation of large language models in medical benchmarking
Source: NPJ Digit Med. 2026 May 18;9:604. doi: 10.1038/s41746-026-02671-w (PMC13448111; doi:10.1038/s41746-026-02671-w)
Supplement: Supplementary file 1 [file 41746_2026_2671_MOESM1_ESM.pdf]

Supplementary Information

Supplementary Note 1. Supplementary Analysis on Construct Validity via Cross-Modality Evaluation

Rationale and Objective

A key challenge in medical benchmarking is ensuring that performance on multiple-choice questions (MCQs) reflects genuine medical knowledge rather than test-taking heuristics or pattern recognition. To address concerns regarding the construct validity of our CAT framework, we conducted a cross-modality validation study.

The objective of this supplementary analysis was to evaluate the correlation between our CAT-based proficiency estimates (MCQ format) and model performance on a generative, open-ended medical QA benchmark and assess the robustness of this correlation using a Dual-Judge Protocol involving both a state-of-the-art generalist LLM and a specialized medical LLM to mitigate potential evaluator bias.

Methodology and Dataset

We utilized the "Medical Knowledge" subset of the LLMEval-Med benchmark [1], comprising 141 high-quality items designed to assess standardized medical knowledge in an open-ended format. Each data entry includes a question, a reference answer authored and refined by medical experts, a prompt simulating real-world usage, and a checklist specifying key content requirements.

Unlike the MCQ task format, the models in this validation phase were presented with the question stem only and were required to generate the answer directly. This format

removes the possibility of guessing based on option elimination strategies. Due to the rapid lifecycle of LLMs, 4 of the original 38 models were deprecated or became inaccessible via API during the revision period (February 2026). Consequently, this analysis was performed on the remaining 34 accessible models. This subset retains the diversity of the original cohort, spanning from small open-source models to large proprietary frontiers.

To mitigate subjective variability in scoring open-ended responses, we adopted the "LLM-as-a-Judge" methodology, employing State-of-the-Art (SOTA) LLMs as automated evaluators. The evaluation includes four core components: Prompt (defining the role and task background), Question, Response, and Reference Answer. During scoring, the judge model utilizes structured scoring prompts to ensure consistent evaluation criteria. These prompts explicitly define scoring dimensions, metrics, and rules, thereby reducing subjective variability. This structured approach minimizes the hallucination or bias often associated with unstructured LLM grading. For detailed item content, prompt designs, and specific scoring examples, please refer to the Supplementary Information of the original LLMEval-Med publication.

Dual-Judge Evaluation Protocol

To ensure the robustness of our scoring mechanism and mitigate potential evaluator bias, we implemented a Dual-Judge Protocol utilizing two distinct large language models with complementary strengths. First, we employed GPT-5.2 as a generalist evaluator. Second, to address domain-specific nuances within the Chinese medical context, we utilized Baichuan-M3 as a specialist evaluator, which is built for clinical decision support. The

temperature parameter was fixed at 0.0 to ensure deterministic outputs. The top-p value was set to 1.0 to allow sampling from the full probability distribution without truncation.

Results

We calculated the Pearson correlation coefficient between the CAT-estimated Theta from the main study and the aggregate accuracy scores from the generative task. As illustrated in Supplementary Figure 1, the cross-modality analysis revealed a robust linear relationship between the CAT-estimated proficiency (θ) and the open-ended generative performance. Notably, the Pearson correlation coefficient remained high and consistent across both evaluators, with GPT-5.2 yielding an r of 0.829 ($p < 0.001$) and Baichuan-M3 similarly yielding an r of 0.831($p < 0.001$) .

Supplementary Figure 1. Cross-Modality Validation of Construct Validity

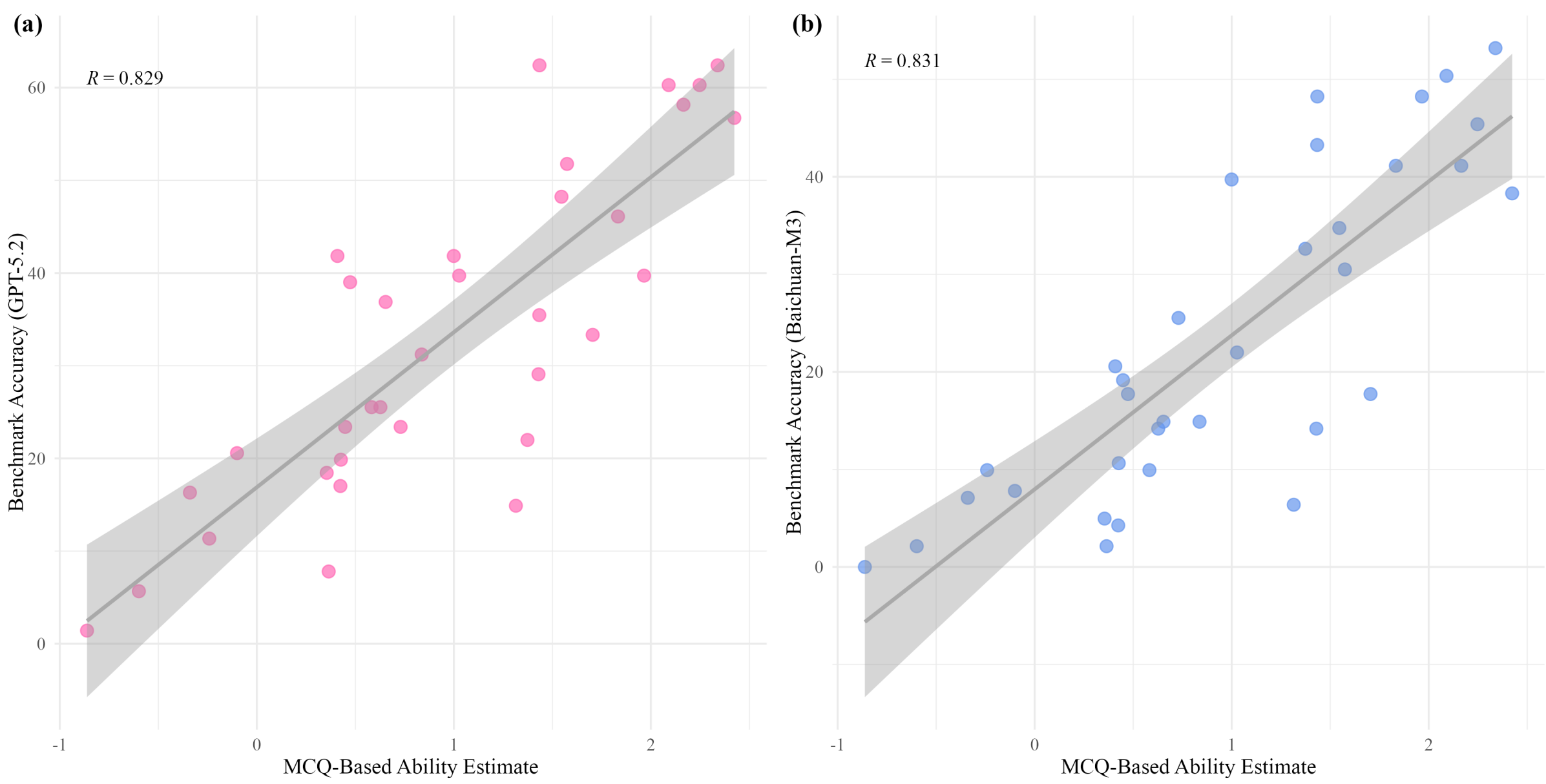

Supplementary Note 2. Technical Details of the Ability Estimation Method

In a Computerized Adaptive Testing system, the estimation of an examinee's latent ability, denoted by θ, is a core iterative step. To ensure robust and stable ability estimates throughout the testing process, our study employed the Expected A Posteriori (EAP) estimation method. EAP is a Bayesian approach that is widely adopted in operational CAT programs due to its superior performance with short tests. The fundamental idea of EAP is to estimate an examinee's ability by calculating the mean of the posterior distribution of θ, given their vector of item responses.

Formally, given an examinee's response vector $\boldsymbol{X} = (x_1, x_2, \cdots, x_j)$ to j items, the EAP estimate of their ability, $\hat{\theta}_{EAP}$, is calculated as the expected value of θ with respect to its posterior distribution:

$$\hat{\theta}_{EAP} = \boldsymbol{E}(\theta|\boldsymbol{X}) = \frac{\int_{-\infty}^{\infty} \theta L(\boldsymbol{X}|\theta) g(\theta) d\theta}{\int_{-\infty}^{\infty} L(\boldsymbol{X}|\theta) g(\theta) d\theta} \quad (9),$$

where $L(\boldsymbol{X}|\theta)$ is the likelihood function of observing the response pattern $\boldsymbol{X}$ given an ability level of θ. Under the assumption of local independence in IRT, this is the product of the probabilities of the individual item responses:

$$L(\boldsymbol{X}|\theta) = \prod_{j=1}^{J} P_j(x_j|\theta) \quad (10).$$

In Eq. (2), $g(\theta)$ is the prior distribution of ability in the population.

In CAT applications, this is typically assumed to be a standard normal distribution, $N(0,1)$, reflecting the distribution of the reference population upon which the item bank was calibrated. The numerator of the EAP formula is the integral of the posterior distribution weighted by θ,, while the denominator corresponds to the marginal likelihood—

the integral of the likelihood weighted by the prior—which serves as a normalizing constant. By incorporating information from the prior distribution, EAP provides a stable and finite ability estimate even under extreme response patterns.

Supplementary Note 3. Technical Details of the Maximum Fisher Information Item Selection Method

In a Computerized Adaptive Testing (CAT) framework, the item selection strategy plays a crucial role in ensuring efficient measurement of examinee ability. Among the most widely used methods is the Maximum Fisher Information (MFI) criterion, which selects the next item based on its information contribution at the current ability estimate.

Formally, under the Item Response Theory (IRT) model, the Fisher information of item *j* at ability level θ is defined as:

$$I_j(\theta) = \mathrm{Var}\left(\frac{\partial}{\partial\theta}\ln P\left(x_j|\theta\right)\right) \quad (11),$$

where $P(x_j|\theta)$is the probability of a particular response to item j at ability level θ. For dichotomous models such as the 2-parameter logistic (2PL) model,

$$P_j(\theta_i) = \frac{exp[\alpha_j(\theta_i - \beta_j)]}{1 + exp[\alpha_j(\theta_i - \beta_j)]} \quad (12),$$

where α_j is the discrimination parameter and β_j is the difficulty parameter. The Fisher information simplifies to:

$$I_j(\theta) = \alpha_j^2\, P_j(\theta)\, [1 - P_j(\theta)] \quad (13).$$

At each stage of CAT, given the current provisional ability estimate $\hat{\theta}$ (obtained, for example, via EAP or MLE), the algorithm computes $I_j(\hat{\theta})$ for all eligible items in the remaining pool. The next item is then chosen as the one that maximizes this information:

$$j^* = \arg\max_{j\in\mathcal{J}} I_j\left(\hat{\theta}\right) \quad (14),$$

where $\mathcal{J}$ denotes the set of items not yet administered.

Supplementary Note 4. Example of Prompt, Question, and LLM Response

Prompt:
Please answer the following single-choice question.
Instructions: 1. Choose the correct option letter from the given choices; 2. Output ONLY the letter of the correct answer. Do not include any explanations or extra text.
Example:
Question:
This is an example question stem where you need to choose the correct answer.
Options:
A.Incorrect answer
B.Incorrect answer
C.Correct answer
D.Incorrect answer
E.Incorrect answer
Answer: C
Now, here is the question you need to answer. Again, output ONLY the letter of the correct answer:
{questions}

The Example of Question:
Question:
A 40-year-old male from Brazil visits his primary care physician after experiencing persistent oral thrush, for which he is prescribed fluconazole. One month later, he reports a dry cough, unexplained night sweats, and breathlessness during physical activity. A chest CT performed upon his hospital admission shows diffuse bilateral ground-glass opacities. Which of the following is the most likely diagnosis?
Options:
A. Bacterial pneumonia
B. Late onset asthma
C. Tuberculosis (TB)
D. Immunosuppression-related pneumonia
E. Pneumocystis jirovecii pneumonia (PCP)

The Example of LLM Response:
E

Supplementary Table 1. Evaluated Models and Their Specifications

Model Name	Type	Category	Access Method
claude-4-sonnet-thinking	Commercial	Reasoning	API
deepseek-r1-0528	Open-Source	Reasoning	API
gemini-2.0-flash	Commercial	Non-Reasoning	API
gemini-2.5-flash	Commercial	Reasoning	API
gemini-2.5-pro	Commercial	Reasoning	API
gemini-pro	Commercial	Reasoning	API
glm-3-turbo	Commercial	Non-Reasoning	API
glm-4-air	Open-Source	Non-Reasoning	API
glm-4.1v-9b-thinking	Open-Source	Reasoning	API
glm-4.5-air	Open-Source	Reasoning	API
glm-4	Open-Source	Non-Reasoning	API
gpt-3.5-turbo	Commercial	Non-Reasoning	API
gpt-4	Commercial	Non-Reasoning	API
gpt-4o-mini	Commercial	Non-Reasoning	API
gpt-4o	Commercial	Non-Reasoning	API
gpt-oss-120b	Open-Source	Reasoning	API
gpt-oss-20b	Open-Source	Reasoning	API
hunyuan-standard	Commercial	Non-Reasoning	API
internlm2-20b	Open-Source	Non-Reasoning	Local Deployment
internlm2-7b	Open-Source	Non-Reasoning	Local Deployment
kimi-k2	Open-Source	Non-Reasoning	API
medgemma-27b	Open-Source	Non-Reasoning	Local Deployment
minimax-m1	Open-Source	Reasoning	API
o1-mini	Commercial	Reasoning	API
o1	Commercial	Reasoning	API
phi-4	Open-Source	Non-Reasoning	API
qwen-max	Commercial	Non-Reasoning	API
qwen2.5-0.5b-instruct	Open-Source	Non-Reasoning	API
qwen2.5-1.5b-instruct	Open-Source	Non-Reasoning	API
qwen2.5-14b-instruct	Open-Source	Non-Reasoning	API
qwen2.5-32b-instruct	Open-Source	Non-Reasoning	API
qwen2.5-3b-instruct	Open-Source	Non-Reasoning	API
qwen2.5-72b-instruct	Open-Source	Non-Reasoning	API
qwen2.5-7b-instruct	Open-Source	Non-Reasoning	API
qwen3-30b-a3b	Open-Source	Reasoning	API
yi-lightning	Commercial	Non-Reasoning	API
yi-medium	Commercial	Non-Reasoning	API
yi-spark	Commercial	Non-Reasoning	API

Supplementary References

1. Zhang, M. *et al.* LLMEval-Med: A real-world clinical benchmark for medical LLMs with physician validation. *Findings of the Association for Computational Linguistics: EMNLP* 4888–4914 (2025). https://doi.org/10.18653/v1/2025.findings-emnlp.263
